# Contemporary Nonpharmacological Strategies to Manage Preoperative Anxiety: Nonsystematic Review

**DOI:** 10.15388/Amed.2024.31.2.17

**Published:** 2024-12-04

**Authors:** Vilma Kuzminskaitė, Agnė Ramaškaitė, Justina Semenkovaitė, Eglė Kontrimavičiūtė

**Affiliations:** 1 Clinic of Anesthesiology and Intensive Care, Institute of Clinical Medicine, Faculty of Medicine, Vilnius University, Vilnius, Lithuania; 2 Faculty of Medicine, Vilnius University, Vilnius, Lithuania

**Keywords:** Preoperative anxiety, nonpharmacological, management, adult, pediatric, Raktažodžiai: priešoperacinis nerimas, nefarmakologinis nerimo valdymas, suaugusieji, vaikai

## Abstract

**Background:**

Preoperative anxiety affects the majority of surgical patients and has a huge impact on their well-being and recovery. Pharmacological anxiety treatment does not always result in the desired effect and occasionally causes side effects. To address this issue, recent research is focused on inventing and studying nonpharmacological anxiety management techniques.

**Objectives:**

To review the diversity of recent nonpharmacological anxiety-reducing methods for adult and pediatric patients and compare their effectiveness and applicability in real-life clinical practice.

**Methods:**

The search was performed using keywords ‘preoperative anxiety’, ‘management techniques’, ‘nonpharmacological’, ‘pediatric’ in PubMed and Google Scholar databases. Full-text original articles or reviews that were published during the last 5 years were selected. Anxiety management techniques were identified and further analyzed for advantages and disadvantages.

**Results:**

Patient education, educational videos and music seem to be the most effective and easy to implement in daily practice for adults. While playing rooms and interactive pop-up books are reasonable to manage preoperative anxiety for pediatric patients.

**Conclusions:**

The choice of nonpharmacological anxiety management methods is diverse. However, the availability and application in reality highly depend on numerous variables, such as costs, time availability, motivation of the medical team, cultural background, and preference of the patient.

## Abstract (it)

**Įvadas:**

Priešoperacinis nerimas vargina daugumą chirurginių pacientų ir daro didžiulę įtaką jų savijautai bei sveikimui. Farmakologinis nerimo gydymas ne visada duoda norimą efektą ir kartais sukelia šalutinį poveikį. Siekiant spręsti šią problemą, naujausiais moksliniais tyrimais daugiausia dėmesio skiriama nefarmakologinių nerimo valdymo metodų išradimui ir tyrimui.

**Tikslai:**

Apžvelgti naujausių nefarmakologinių nerimą mažinančių metodų, skirtų chirurginiams pacientams – suaugusiems ir vaikams, įvairovę ir palyginti jų veiksmingumą bei pritaikymą realioje klinikinėje praktikoje.

**Metodai:**

Paieška atlikta pagal raktinius žodžius „priešoperacinis nerimas“ (‘preoperative anxiety,’), „valdymo būdai“ (‘management techniques’), „nefarmakologiniai“ (‘nonpharmacological’), „vaikų“ (‘pediatric’) *PubMed* ir *Google Scholar* duomenų bazėse. Buvo atrinkti viso teksto originalūs straipsniai arba apžvalgos, paskelbti per pastaruosius 5 metus. Nustatyti nerimo valdymo būdai ir toliau analizuoti jų privalumai ir trūkumai.

**Rezultatai:**

Pacientų mokymas, mokomieji vaizdo įrašai ir muzika atrodo veiksmingiausi ir lengvai pritaikomi kasdienėje suaugusiųjų praktikoje. Tuo tarpu žaidimų kambariai ir interaktyvios iššokančios knygos yra tikslingos vaikų pacientų priešoperaciniam nerimui valdyti.

**Išvados:**

Nefarmakologinių nerimo valdymo metodų pasirinkimas yra įvairus. Tačiau jų prieinamumas ir taikymas labai priklauso nuo daugelio kintamųjų, tokių kaip išlaidos, laiko galimybės, medicinos komandos motyvacija, kultūrinė aplinka ir paciento pageidavimai.

## Introduction

Anxiety before surgery and anesthesia is a very widespread problem in perioperative medicine, affecting up to 75% of patients admitted for the surgical procedure [1]. High incidence of preoperative anxiety and its negative implications on patients’ physiology, recovery and satisfaction inspired decades of research on anxiety management [2,3].

Pharmacological measures to relieve anxiety, especially benzodiazepines, are cheap and usually do not lead to serious side effects when used in small doses. However, some latest research fails to prove significant anxiolytic effects of these medication [4]. Therefore, there is a shift towards nonpharmacological techniques. Multiple methods to manage preoperative anxiety have been described in previous literature. The efficacy of many of those methods has been proven in clinical trials. But the question arises about how widely these techniques are actually implicated in everyday clinical practice.

In this nonsystematic review we present the latest trends in nonpharmacological preoperative anxiety management and provide some insight into effectiveness, applicability and advantages and disadvantages of identified techniques. Additionally, we introduce the reader to several anxiety management strategies from other fields of medicine that could be applied to perioperative setting as well.

## Methods

We have reviewed PubMed and Google Scholar databases for full-text original articles or reviews using the keywords ‘preoperative anxiety,’ ‘management techniques,’ ‘nonpharmacological,’ ‘pediatric,’ and their combinations during the period of 5 years (2018–2023). The results from the primary literature search were filtered and duplicates were removed.

Preoperative anxiety measures were then identified, and deeper literature analysis for advantages and shortcomings of each of the described techniques was performed.

## Preoperative anxiety management techniques

### 1. Preoperative patient education

Providing patients with information about the anesthesia procedure is a necessary step of patient care before every surgery. It is extremely important to understand how that information should be delivered to keep the anxiety levels to a minimum and to make sure that the patient is capable of understanding information that has been provided. A previous study [5] found that the largest proportion of patients (54%) supported the leaflet with detailed information explaining the procedures.

On the other hand, the study predicted that not all patients might need additional information because expanded amount of knowledge could also have an opposite effect of increasing the anxiety levels in some groups of the patients: 12% of respondents chose to know less rather than more, to protect themselves from additional stress. However, it was noted that regardless of this finding, a sufficient amount of knowledge needs to be provided for the purpose of obtaining an informed consent from the patient [5]. However, providing only the necessary facts rather than more in-depth information additionally could result in consultation time being saved [6]. Other studies also acknowledge that patients require different amounts of preoperative education, therefore individualized anxiety management should be available for patients and their choice must be respected [6–8].

The need for information before providing it could be assessed in patients, similar to how patients are being physically examined before any medical procedure. The study of Wongkietkachorn et al. (2018) concluded that patients, who received education based on their needs, benefited from this approach by having their anxiety significantly decreased and their overall satisfaction with the hospital experience was better compared to the traditional education group. Yet, it is important to notice that the need for information might differ not only among patients in the same population, but also among different countries and cultures [1,4].

### 2. Telemedicine

Telemedicine is another form of information delivery that has been recently studied [9]. Consulting patients via the telephone or video-call platforms is becoming increasingly common, especially in the outpatient setting. Conveying information through telephone allows an increased focus on the content rather than the surroundings or the nonverbal aspects of the conversation.

Even though the study of Gibas et al. (2022) did not find a relieving effect on patients’ preoperative anxiety, there were no side effects from using this information providing technique. On the contrary, telephone consultations added numerous benefits compared to the preoperative consultations in person at the outpatient clinic. The traveling time was saved, which is extremely important for people living in distant or rural areas, and for those who have difficult access to transportation.

Telephone consultations did not have a fear and anxiety increasing effect, which makes them as good as face-to-face consultations, if not better [9]. In our opinion, this information delivery method is especially valuable in the after-pandemic period, as it protects patients from contracting infections during a hospital visit.

### 3. Video information

Several studies have recently examined the impact of patient-information videos on anxiety levels before surgery. As evidence shows, this form of information delivery can significantly reduce patients’ anxiety when used during the preoperative visit [8,10]. One of the studies [10] found that anxiety levels for the intervention group even continued dropping further until the time of the surgery. In addition to reduced anxiety, the hemodynamic parameters of the patients fluctuated significantly less from the measured baseline in the intervention groups [10,11], potentially reflecting reduced anxiety and calmer emotional state. Educational videos also result in improved hospital experience compared to only verbal explanations from the doctor [8]. However, some beneficial results could be affected by benzodiazepine premedication or double effect of the video and the additional verbal briefing provided by the anesthesiologist before the surgery [8].

Like conventional patient education, the content of the information provided in the video matters. Ahmed et al. predicted that showing videos with different content and focus could possibly result in a range of different outcomes [8]. Some studies focused on the explanation and visualization of spinal anesthesia technique [10], different types of anesthesia and its risks [9], explained the surgery and all the necessary patient care steps, as well as the healing process [11]. Contrary to the suggested opinion [8], these studies were also able to successfully conclude that the videos caused an anxiety relieving effect, despite the content being more graphic, and detail-focused.

Video information not only relieves preoperative anxiety [8,10,11], but provides multiple additional advantages. First, it is an inexpensive and efficient method, which can provide standardized information for all patients. Second, it is a quick and simple way to improve hospital service and is extremely helpful when treating large amounts of patients in busy hospitals [8,10]. Third, nowadays patients tend to search for information and educate themselves using the Internet. Lack of specialist knowledge and inability to distinguish trustworthy sources from unreliable ones might confuse the patient and cause unnecessary concerns and increase anxiety levels. A video provided by the hospital could prevent this problem [8]. Finally, this form of patient education provides the opportunity to explain certain aspects of anesthesia and surgery experiences visually and in ways that would not be possible during a doctor–patient consultation [8]. On the other hand, no individualized information regarding specific patient risks or conditions could be provided by mass-production videos.

Some studies also test dual anxiety relieving techniques. Combination of video information and music [11] or telemedicine [9] can successfully lessen preoperative anxiety as well.

### 4. Interactive techniques and play

A couple of studies examined child-friendly information delivery methods for pediatric patients. A pop-up book [12] was suggested and its effectiveness in reducing anxiety was tested. The pop-up book provided more in-depth knowledge about anesthesia and surgery compared to standard information given for patients, and interactively answered the most frequent questions children might have about usually frightening anesthesia and surgery experience. The interactive book itself was perceived as entertaining and better adapted for children’s comprehension abilities rather than the prepared video and consultations with doctors. This child-focused technique resulted in overall more positive expectations of the hospital and anesthesia experience, more trust in child–doctor relationship, and was able to significantly reduce children’s anxiety before surgery and postoperative pain.

Conversely, a similar study of Garcia de Avila et al. [13] analyzing the effect of preoperative children education through a comic book failed to show reduced anxiety levels in pediatric patients. The authors speculated that the lack of desirable outcome could be due to the intervention being performed on the same day as the surgery and suggested that the anxiety-reducing preparations should be started as early as possible, preferably from the day when the operation is scheduled. However, the previously described pop-up book intervention [12] was also carried out on the same day as the surgery and succeeded in proving positive outcomes. Interactive features, such as moving characters, lifting and moving various flaps to reveal more information, scratch-and-sniff stickers and more, in the pop-up book offers more engagement compared to comic book with no such features.

One of the studies [14] investigated preoperative activities in a playing room such as reading, drawing, toys and games, compared to treating anxiety with medicine. The children who participated in these activities were significantly less anxious than the control group during the whole waiting time and during the induction of anesthesia, while anxiety in the control group kept increasing until the beginning of surgery. The playing room was especially effective in children who were at least 7 years and older.

Another interesting approach to anxiety prevention for children – exposure to anesthetic mask by parents [15]. The authors focused on the time and frequency of familiarizing the child with the mask. The results showed that this technique is not only effective in reducing preoperative anxiety, but also revealed that exposure to mask and practice instructions on the morning of the surgery was more effective than early intervention the week before the surgery, which showed similar effectiveness as the control group.

### 5. Operating room tour

Showing the operating room before surgery could be one of the ways to reduce anxiety in surgical patients. In one study, the tour was given one day before the surgery and later 10 mg of oral diazepam was administered to both the intervention and control groups before sleep. The levels of anxiety shifted from high before having the tour to moderate the next day before the surgical procedure. Decreased hemodynamic parameters, such as blood pressure and heart rate were also evident [16].

Similar techniques are more widely applied within the pediatric population. In addition to viewing the operation room, one of the studies [17] provided children with an opportunity to get acquainted with a simulated anesthesia induction. This method proved to be successful not only in significantly reducing the patients’ anxiety but also their parents’ anxiety, compared to standard preanesthesia consultation. Another study [18] experimented with a playful presentation of equipment used during anesthesia while the children were on the preoperative tour. Compared to midazolam group, this nonpharmacological approach reduced children’s and their parents’ anxiety significantly better. Various studies show that this technique of anxiety management is highly effective for patients of different age groups. Familiarization with an unknown environment is beneficial not only for the emotional state of the patients and readiness for the surgery but also for the patients’ overall satisfaction and less often postponing of the surgery [16,17].

Then again, it takes a significant amount of time and additional staff to lead the patients through the operating room in preparation for surgery. In some surgery cases the patients might need to be admitted to the hospital one day earlier than otherwise necessary and hence the costs of nursing care increase greatly. For this reason, this highly effective method might be suitable in smaller hospitals with lower numbers of daily surgeries and patients.

### 6. Virtual reality

Virtual reality (VR) offers immersive experience with interactive sensory stimuli and has been used to treat anxiety disorders for years [19]. It could serve as another form of providing information to patients or as an alternative form of real operating rooms tours. Grab et al. investigated the effect 3D-printing and virtual reality has on preoperative anxiety in cardiac surgery patients [20]. Digital models were created and used during preoperative education to illustrate the principles of cardiac surgery. Significant anxiety reducing effect was observed when patients were educated using VR. Visual experience was favorably met by patients and significantly improved patients’ understanding of the procedure.

According to the systematic review and meta-analysis by Koo et al. VR seems to have a superior anxiolytic effect in pediatric patients rather than adults [21]. Here timing and duration of VR experience may be the key to successful anxiety management. However, VR techniques require special equipment and additional time to set it up [20]. Certain patients might find it difficult to use the controllers due to cognitive or physical status (dementia, significant tremor, paresis, etc.). Also, virtual reality may have some minor, yet undesirable side effects, such as uncomfortable headsets [19], dizziness [20], and virtual reality sickness, which could be avoided by limiting the duration of VR sessions [22].

### 7. Music therapy

Preoperative listening of music for anxiety management is currently getting a lot of attention among doctors and researchers in various countries. A study conducted in Turkey [11] revealed that 30-minute-long sessions of classical Turkish significantly lower anxiety levels and hemodynamic parameters compared to the control group. However, additional preoperative education was also provided to the intervention group, which makes evaluating the individual effectiveness of music problematic.

Another study compared the difference between self-selected and predetermined music on patients’ anxiety [23]. After a 20-minute music session both groups of patients felt significantly less anxious, and no significant difference in anxiety levels between the two groups was observed. However, it is worth mentioning that this study included female patients only, as the interventions were applied before gynecological surgeries, therefore the results cannot be accurately applied to the whole population.

Music therapy was also tested with children [24]. Listening to soft music or songs for kids for 30 minutes before surgery significantly decreased anxiety and hemodynamic parameters for pediatric patients. In addition, this intervention increased anesthesia induction compliance, improved children’s mood, and the atmosphere in the operating room.

As was demonstrated in these studies, music seems to be an especially effective, practical, and easy to apply intervention in managing preoperative anxiety of all age groups. Yet, this intervention requires a significant amount of time without interruptions, which could be difficult to achieve in the hospital environment where interaction with medical staff and other patients is frequent. The patients could be educated on the positive and anxiety relieving effects of music on the day when the surgery is scheduled. Keeping in mind that self-selected music, regardless of the genre, is just as effective as any specially prepared relaxing playlist [23], patients could be advised to choose and prepare a playlist of music they enjoy. On the day of the surgery, the music could be listened to whenever patients have time or feel anxious while waiting for the operation.

### 8. Aromatherapy

Several studies showed that using aromatherapy as additional intervention in the preoperative period can decrease anxiety scores. Therapeutic inhaled essential oils (TIEO) are the intranasal inhaled vapors of natural and plant essences, which triggers olfactory receptors and sends nerve stimulus to the brain leading to calming psychological process.

The effect of TIEO therapy on preoperative anxiety was tested on women undergoing gynecological surgeries. Patients who used aromatherapy reported lower levels of anxiety. In addition, patients found the scent of TIEO enjoyable, which provided a nice distraction while waiting for their surgery in the preoperative room [25].

Another study conducted in the USA evaluated how the use of lavender aromatherapy skin patch helps to manage preoperative anxiety in women scheduled for elective breast cancer surgery. Patients used skin patches from 26.7 to 89.5 minutes. Although no optimal duration for the skin patch use was determined, it was noticed that this method reduces not only anxiety, but also physiological signs of anxiety as well [26].

Systematic evidence from meta-analysis of 750 patients also prove aromatherapy to be effective in reducing preoperative anxiety and could be used as a complementary measure in anxiety treatment [27]. Yet, the authors conclude that the strength of evidence is doubtful due to methodological limitations of the studies.

Aromatherapy manages preoperative anxiety by creating a pleasant environment. On the other hand, some unfavorable effects such as sleepiness, nausea and skin irritation of using topical essential oils were reported [28]. No adverse effects were reported using TIEO. However, a few cases of melioidosis after the exposure of aromatherapy room spray should keep alertness of medical staff [29]. Crossover study findings also show that aromatherapy may increase behavioral and psychological symptoms of dementia severity during washout period and the postoperative follow-up evaluation [28].

### 9. Phytotherapy

Some plants are well-known for their calming effects and could be used for preoperative anxiety management. Study conducted in Iran assessed anxiolytic effect of bitter orange *(Citrus aurantium)* blossom in preoperative period. Patients who were premedicated with *Citrus aurantium* blossom distillate had lower anxiety levels than placebo group. However, *Citrus aurantium* interferes with drugs related to anesthesia such as antihypertensive, sedative and antiemetic agents and needs to be used with caution [30].

Other studies examined the anxiety-reducing effect of passionflower (*Passiflora incarnata*) in patients with neuropsychiatric disorders. The use of the passionflower extract effectively reduced anxiety level for patients with generalized anxiety disorder compared with oxazepam. Other studies also report decreased anxiety after the administration of passionflower syrup before medical interventions such as spinal anesthesia, dental procedures or surgery [31]. The authors suggest that the use of passionflower is a safe and effective method reducing preoperative anxiety and has similar effect compared with the pharmacological anxiety-reducing forms [31].

Another widely used plant in phytotherapy is lemon balm (*Melissa officinalis)*. It is well tolerated and only occasionally causes headache, abdominal pain, nausea, vomiting [21].

### 10. Other techniques

Finally, it is worth mentioning some less studied but interesting and innovative anxiety reducing methods. Some authors have analyzed how different kinds of massage affect preoperative anxiety. For example, one study [32] focused on extremity massages. Hand and foot massages were compared with one another and also with a placebo (holding patient’s hand before surgery). In contrast to placebo, both hand and foot massages were helpful in significantly reducing anxiety with no significant difference between the two techniques.

A systematic review on auricular stimulation for anxiety management also demonstrates similar positive effects [33]. Methods such as auricular acupuncture, electroacupuncture, and acupressure were all successful in reducing patients’ anxiety in comparison to no intervention and the effect was comparable to pharmacological anxiety treatment. Emotional Freedom Technique (EFT) that involves stimulation of acupressure points by tapping or rubbing while shifting the focus on the stressful situation shows promising results in laparoscopic cholecystectomy patients [34].

A recent meta-analysis including 8 studies and 1242 patients concluded hypnosis to be effective when alleviating preoperative anxiety and postsurgical pain in breast cancer patients [35]. Similar effects were found in onco-gynecological [36] or dental surgery; however, a certified specialist is needed to avoid risks associated with hypnosis [37].

Evidence exist that patient interaction with therapy animals relieves stress, pain, and anxiety in multiple healthcare settings and could reduce posttraumatic stress symptoms in the long term as well [38–40]. A controlled trial of Kline et al. demonstrated the effectiveness of canine therapy when reducing self-reported anxiety levels in emergency department patients [41].

Even 15 minutes of exposure to assistance dogs relieves anxiety making this technique little time consuming and attractive option for medically stable patients. However, additional canine therapy specialists, and dogs trained by accredited assistance dog programs are required. This therapy would not be suitable for patients with allergy for dogs or cynophobia. Similarly, application of canine therapy is limited in areas with strict infection-control requirements.

Cognitive behavioral therapy (CBT) includes several strategies and techniques to combat anxiety maintaining thoughts and behaviors [42]. Yang et al. proved cognitive and behavioral interventions to be useful in functional endoscopic sinus surgery where it reduced preoperative anxiety and cortisol levels and was associated with improved outcomes [43]. Cognitive behavioral play therapy has been applied in pediatric population as well [44]. It resulted in reduced preprocedural anxiety and was more efficient than audiovisual distraction.

The downside of CBT is that the training requires a couple of hours, and repetitive practice sessions are needed to ensure the effect. Telephone or internet means are also used as an equivalent, accessible, and cost-effective means of delivery for CBT interventions as person-to-person contact is [45].

Mindfulness is described as nonjudgmental and nonreactive awareness of the present moment [42]. Distancing the patient from negative thoughts could reduce anxiety and postoperative pain [46]. Mindfulness is a cheap method, and the use of internet and virtual technologies could ever further alleviate its implementation into clinical practice, but the timing and standardization of the intervention could be challenging [47].

In general, mindfulness is a safe method. However, increased awareness sometimes can lead to increased perception of discomfort. Nevertheless, it is considered as a mild side effect, and serious adverse events are rare and occurring with intensive practicing [48]. Mindfulness may not be an appealing option for patients who are not ready to take the reflexive approach of their thoughts or emotions.

The characteristics, including advantages and disadvantages, of the previously mentioned anxiety management techniques are summarized in Table 1.

**Table 1:** The summary of characteristics of anxiety management techniques

	Time consumption	Need for add. staff	Add. costs	Applicability in pediatric patients	Easy impleme-ntation	Advantages	Disadvantages	Comments
**Preoperative patient education**	++	+/-	-	Age dependent	+	Relatively inexpensive	Possibly time consuming May increase anxiety in some patients My not be suitable for cognitively impaired patients	Need for future research to quickly identify which patients are information seekers, what information should be provided and what is the best timing of education Standard information could save time and costs Effectiveness may be influenced by cultural factors
**Telemedicine**	++	+/-	-	Age dependent	+	Time saving (physician and patient compared to in person consultations) Individual information	Internet connection required	Especially useful for patients living in distant areas, in case of pandemic or restricted (physical/transportation) access
**Video information**	++	-	+/-	Age dependent	++	Single investments to prepare the material May be watched from home No additional staff Patients may skip certain information on the video if not willing to know “too much” or replay more relevant information	Standard information may not address individual patient needs	Improved patient experience compared to verbal information only Future research is needed on determining the type of content material that is the most effective in reducing anxiety
**Interactive techniques**	++	-	+/-	+	+	Entertaining for children Standardized simplified information	Additional time and equipment	Communication and information provision techniques must be age-tailored in pediatrics Contradictory results due to variety of interactive techniques used: some may be more effective that the others
**Operating room tour**	+++	+	+	+	-	Introduction to the perioperative environment increases the feeling of safety and control Individual information can be provided Suitable for all ages	Costly and time-consuming Interrupts the schedule of the operating room	More suitable for smaller institutions Group tours are possible to save costs
**Virtual reality**	++	+	+	+	+/-	Visual information Engaging and attractive Suitable in pediatric patients	Special equipment required More effective in children May not be suitable for patients with significant cognitive and physical diseases Side effects (e.g., VR sickness)	More data is needed to find out the VR effects in children with high level of anxiety Unclear timing Useful to reduce postoperative pain as well
**Music therapy**	++	-	+/-	+	++	Easy to implement Minimal to no expenses	Time consuming Quiet environment is needed	Preselected or patient-chosen music is equally effective
**Aromatherapy**	+++	+	+	-	-	Mostly pleasant process Could be applied as group therapy	Time consuming Special equipment and room is needed Side effects are possible (e.g., nausea, allergic reactions)	Acceptability and effectiveness of the technique may be culturally dependent
**Phytotherapy**	+++	+	+	-	-	Effective	Additional staff Time consuming Possible drug interactions and side effects (e.g., headache, nausea, allergic reactions)	May interrupt with preoperative fasting guidelines Unclear effectiveness when different severity of preoperative anxiety
**Massage, acupressure, EFT**	+++	+/-	+/-	Age dependent	-	Pleasant and relaxing	May require special equipment (e.g., electroacupuncture)	Few studies have been done Variety of techniques available
**Hypnosis**	++	+	-	-	-	May reduce postoperative pain as well Safe	Certified specialist is required	Not suitable for hypnosis-resistant patients
**Therapy animals**	+	+	+	+	+/-	Relatively short session are sufficient	Specialists and trained animals required Not suitable for allergic patients or patients with animal phobias	Future studies could investigate the effectiveness in preoperative setting May be incompatible with infection-control requirements
**CBT**	++	+/-	-	+	+/-	Suitable for different ages	Long training and repeated sessions are required	Could be used via telephone or internet
**Mindfulness**	+	-	-	Age dependent	+/-	Cheap Safe	Training required May increase discomfort in certain patients	Implementation could be facilitated by using internet or virtual technologies

## Discussion

Nowadays, as the modern medical world is acknowledging the importance of managing patients’ anxiety, there is a wide range of studies being released that focus on investigating various nonpharmacological preoperative anxiety management techniques. Most of them were proven to be effective and were favorably accepted by the patients.

Having such a wide range of techniques to choose from leaves us with an important task – to evaluate which ones are effective enough and easiest to implement in daily hospital practice. To do this, we chose to assess the costs, the duration of time needed to implement a specific technique, possible side effects, and the need for additional staff. We believe that the best anxiety-reducing methods must also be practical enough to be used daily without requiring a lot of additional effort.

Preoperative patient education and educational videos would seem to be the techniques that match the criteria described above the best and complement each other. The need for extra information could be identified by the anesthesiologist during the preoperative consultation and a video or a leaflet could then be shown to those groups of patients who prefer to know more, avoiding the overflow of information for those who prefer to know less.

We also believe that listening to music preoperatively or even during the procedures when local or regional anesthesia is applied is one of the most realistic and easy to implement anxiety-managing techniques that was proven to be effective [11,23,49]. Since listening to self-chosen music is as effective as listening to predetermined music [49], patients could be instructed to prepare a playlist of their favorite music and be prepared to listen to it before surgery for anxiety management. This kind of patient self-preparation would easily save time, costs and omit the need for additional staff.

On the other hand, techniques such as operating room tours, aromatherapy, phytotherapy, massages, and acupuncture would require a relatively large amount of time and additional expenses along with staff that are well-trained and have specialist knowledge in these procedures. Therefore, even though these interesting and effective methods were gladly received by patients, they do not seem to be realistic and practical enough to be applied as routine patient care practice.

When it comes to pediatric patients, playing rooms, interactive pop-up books, and VR explaining anesthesia and surgery seem like the best and most effective options for children. It is likely that some children’s hospitals already have playing rooms so no additional time or expenses would be needed to manage anxiety while waiting for surgery in those hospitals. Educational interactive books could be used by anesthesiologists during preoperative consultations to reduce children’s anxiety and help them understand what is going to happen better. The extra time and cost would be minimal and there would be no need for additional staff. On the contrary, it might be a little unrealistic to expect wide application of the VR method in current times. However, it is a quickly developing and improving technology and is highly effective in managing children’s anxiety [21]. Soon enough this intervention could become cheaper and more available for pediatric patients around the world.

## Conclusions

When considering all the nonpharmacological anxiety-reducing techniques, it is certain that their availability greatly depends on the finances of the hospital and the specific country. Also, most importantly, on the motivation of doctors to research and apply these methods in their daily practice as well as on the willingness of the institution to encourage them to do so. Preoperative anxiety is a complex problem with varying degrees of intensity and different underlying causes tightly related to cultural and religious background. Some interesting and unusual techniques could be more available and common in certain countries and cultures of the world than others. Likewise, patients from various backgrounds might prefer different anxiety-reducing interventions. Due to all of these reasons, it is challenging to conclude which preoperative anxiety management technique is indeed superior to others.

Therefore, we suggest that the open-mindedness of the physician and hospital administration is the key necessary to successful management of preoperative anxiety.

## Data Availability

The manuscript is of the review nature. Therefore, there is no data or materials collected to be available.
